# Automated Collateral Scoring on CT Angiography of Patients with Acute Ischemic Stroke Using Hybrid CNN and Transformer Network

**DOI:** 10.3390/biomedicines11020243

**Published:** 2023-01-17

**Authors:** Hulin Kuang, Wenfang Wan, Yahui Wang, Jie Wang, Wu Qiu

**Affiliations:** 1 Hunan Provincial Key Lab on Bioinformatics, School of Computer Science and Engineering, Central South University, Changsha 410083, China; 2 School of Life Science and Technology, Huazhong University of Science and Technology, Wuhan 430074, China

**Keywords:** acute ischemic stroke, collateral scoring, CT angiography, hybrid CNN and Transformer

## Abstract

Collateral scoring plays an important role in diagnosis and treatment decisions of acute ischemic stroke (AIS). Most existing automated methods rely on vessel prominence and amount after vessel segmentation. The purpose of this study was to design a vessel-segmentation free method for automating collateral scoring on CT angiography (CTA). We first processed the original CTA via maximum intensity projection (MIP) and middle cerebral artery (MCA) region segmentation. The obtained MIP images were fed into our proposed hybrid CNN and Transformer model (MPViT) to automatically determine the collateral scores. We collected 154 CTA scans of patients with AIS for evaluation using five-folder cross validation. Results show that the proposed MPViT achieved an intraclass correlation coefficient of 0.767 (95% CI: 0.68–0.83) and a Kappa of 0.6184 (95% CI: 0.4954–0.7414) for three-point collateral score classification. For dichotomized classification (good vs. non-good and poor vs. non-poor), it also achieved great performance.

## 1. Introduction

Stroke is one of the leading causes of death and disability in adults worldwide, and ischemic stroke accounts for the majority, mainly due to atherosclerosis. Currently, the most effective treatments for acute ischemic stroke (AIS) are intravenous thrombolysis and endovascular therapy (EVT) within a suitable time window [1]. To assess the infract core for AIS patients, there are two classical neuroradiological methods: infarct volume size and location estimation (i.e., infarct segmentation) and the Alberta Stroke Program Early CT Score (ASPECTS). For manual infract segmentation, neuroradiologists often visually quantify and locate the infract core via screening all slices of computed tomography (CT) scans or diffusion weighted imaging (DWI) scans to determine which voxels can be infarcts [2]. ASPECTS is a scale to better quantitatively evaluate the affected status of 10 regions in the middle cerebral artery and is implemented via screening scans to determine whether a region contains infarcts [3]. These two types of methods evaluate the ischemic infarct core from different scales, and they are both used in the diagnosis of AIS and help to select patients who are suitable for endovascular treatment [4,5,6,7]. However, the above methods only reflect the infarction core situation and ignore the role of collateral circulation in the treatment of AIS patients. Previous studies have shown that the quality of collateral circulation reflects to a certain extent the amount of brain tissue that can be salvaged [8] and AIS patients with good collateral circulation can receive better clinical outcomes after EVT [9,10]. Therefore, evaluating the collateral circulation status score (i.e., collateral scoring) is necessary and helpful to select patients who may have a good prognosis after EVT.

In the clinical practice, collateral scoring is generally based on visual scoring via screening medical image scans such as CT angiography (CTA) and CT perfusion, which depends on the experience level of the radiologist, resulting in great observer variability [11,12]. Additionally, it is tedious and time-consuming for clinicians to score visual collateral circulation status, which may result in inefficient diagnosis and patient selection. Therefore, an objective and accurate automated collateral scoring method is desired in AIS clinical practice.

In recent years, radiomics and deep neural networks have been widely used in image analysis tasks [13,14,15,16,17,18]. Several methods that are based on radiomics and deep learning have also been used for automated collateral scoring [19,20,21,22]. For example, Su et al. [20] used a convolutional neural network (CNN) to segment blood vessels in the left and right middle cerebral artery (MCA) regions of the brain, and then several quantified features were extracted on the segmented vessels and input into several machine learning approaches to achieve collateral scoring. Some commercial software such as StrokeViewer has also been evaluated for automated collateral scoring in some studies [23,24]. Although these methods can achieve automated scoring of collateral status, most of them are based on the results of vessel segmentation. The performance of vessel segmentation greatly influences the final collateral scoring performance. In this study, we explore vessel-segmentation free methods to potentially improve the collateral scoring performance.

Currently, Transformer [25] has been used in many image analysis tasks due to its excellent ability to capture long-range dependencies. Additionally, how to combine Transformer and CNN to make full use of their advantages has also attracted more and more attention in the field of medical image analysis. For example, Cheng et al. [26] developed a multi-task hybrid CNN–Transformer encoder for isocitrate dehydrogenase prediction and glioma segmentation. Inspired by the fact that CNN is good at learning local representations and Transformer can capture global representations well, we hypothesized that using a hybrid network of Transformer and CNN can score collateral status more accurately and efficiently. We aimed to design a hybrid CNN and Transformer-based vessel-segmentation free method for automating collateral scoring on CTA scans of AIS patients.

## 2. Materials and Methods

### 2.1. Data Acquisition

We collected single-phase CTA scans of 154 AIS patients from Xiangya hospital, Changsha, Hunan. The patient inclusion criteria included: (1) The CTA scans of patients covered the whole brain without severe motion artifacts; (2) expert readings of collateral scores were available.

The sizes of the acquired CTA scans were 512 × 512 × S, where S ranged from 124 to 414. The collateral score is a relatively simple three-point scoring system, i.e., good (score = 0), intermediate (score = 1) and poor (score = 2). Good collateral indicated that the degree of collateral filling was 100%, intermediate collateral indicated that the degree of collateral filling was >50% and <100%, and poor collateral indicated that the degree of collateral filling was ≥0% and less than 50%. All CTA images were separately assessed by two radiologists with more than 10 years of experience, and images that disagreed between the two radiologists were assessed by a third physician with more than 15 years of experience, and they then came to a consensus score. Of all 154 patients, 69, 52 and 33 patients had collateral scores of 0, 1 and 2, respectively.

### 2.2. Image Preprocessing

**Figure 1:**
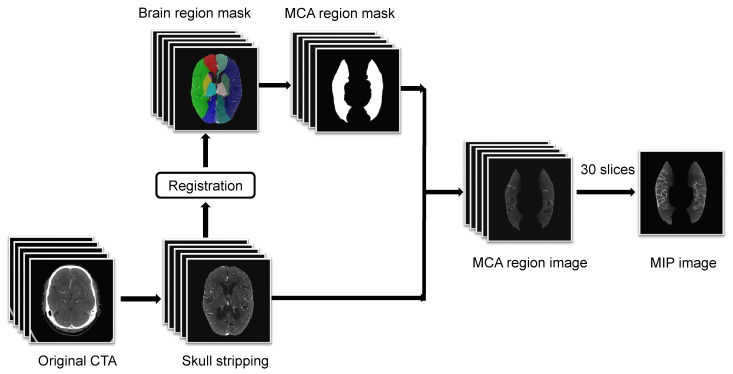
Image preprocessing.

Figure 1 shows the image preprocessing steps used in this study. Skull stripping was first applied, as the high-brightness pixels of the skull had a severe interference in vessel recognition. We used the method in [27] to achieve skull stripping on the acquired CTA images. This method extracts brain within images on a slice-by-slice basis through thresholding combined with convex optimization iterations.

The second preprocessing step was atlas-based registration for MCA region mask generation. We registered the standard brain region atlas onto each CTA scan to obtain the brain region masks of each CTA scan. We generated the MCA region mask from the obtained brain region mask by binarization where voxels that belonged to MCA were set to 1 and the rest were set to 0.

**Figure 2:**
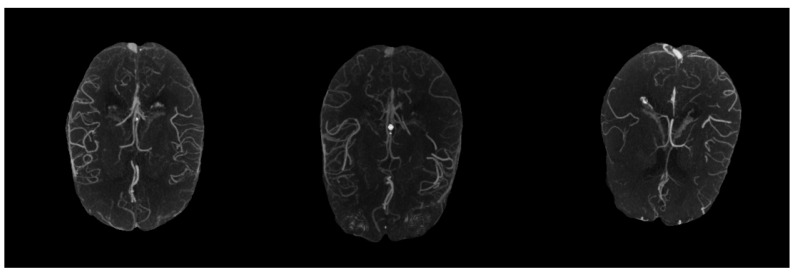
Examples of maximum intensity projection (MIP) images from 3D CTA image with different collateral scores. The left one is good collateral. The middle one is intermediate collateral. The right one is poor collateral.

Because the vessel voxels in 3D CTA scans generally had high intensities, maximum intensity projection (MIP) that projected 3D CTA scans to two-dimensional images could well display the degree of stenosis, dilation and filling defect of vessels [21]. Figure 2 shows visualized MIP results of CTA scans with different collateral scores. As shown in Figure 2, after MIP on the whole 3D scans there were significant differences in the degree of collateral filling in patients with different scores, but there existed some interference outside the MCA regions. Additionally, since the vasculature capable of assessing the status of collateral extended from the skull vertex to the circle of Willis, too much irrelevant vasculature would easily interfere with the scoring [28]. Thus, in order to clearly show the vessels in the MCA arterial tree in the left and right hemispheres and reduce the interference from varying numbers of slices, we only took the 30 most-cranial axial slices from the circle of Willis and multiplied the acquired 3D CTA images with the generated MCA masks before performing MIP in the final preprocessing step.

### 2.3. Collateral Scoring Based on MIP Images

Due to the class imbalance of the samples, we performed data augmentation to alleviate the imbalance. First, we randomly upsampled the poor and intermediate classes with a smaller sample size to the same size as the good class via copying. However, simply copying a large number of images might lead to an overfitting problem. In order to avoid this problem, we implemented operations such as rotations with small angles and shifts without changing the characteristics of CTA scans to ensure the validity of the augmented samples.

**Figure 3:**
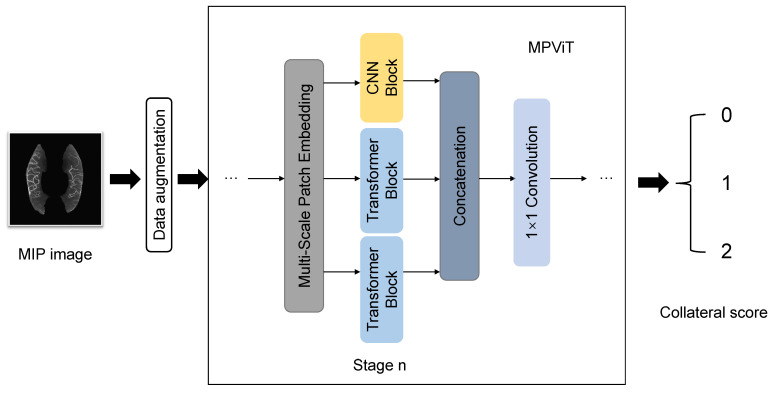
The framework of our designed hybrid CNN and Transformer network for collateral scoring.

From Figure 2, we found that the vessel regions became more salient in MIP images, making it possible to design a vessel-segmentation free method using MIP images. After performing data augmentation on the obtained MIP images, we used a hybrid deep learning model: the MPViT proposed in [29], which combines Transformer and CNN to capture global and local information at the same time. MPViT first processed the input images through a convolution-based stem module to change the image size to 128 × 128. Then, it utilized four stages to learn effective representations for collateral scoring. Each stage consisted of a multi-scale patch embedding based on convolution with different strides, several Transformer-based blocks (the number of blocks is adjustable) to capture global information, a CNN-based block, and a concatenation layer followed by a 1 × 1 convolution layer to effectively combine features from Transformer and CNN blocks. The classification results were achieved by a global average pooling and a linear layer. For more technical details of MPViT, please refer to [29]. Figure 3 shows the framework of our designed hybrid CNN and Transformer network for collateral scoring.

Additionally, in order to verify the advantages of the MPViT method over other automated scoring methods, we also applied three methods on the same MIP images. The first one was a radiomics-based method where we extracted radiomics features of MCA regions that were fed into support vector machine (SVM) for automated collateral scoring. The extraction of radiomics features was done through the software package Pyradiomics (https://pyradiomics.readthedocs.io/en/latest/, accessed on 1 December 2022). Since the extracted features were not all effective, we used the LASSO method for feature selection to avoid feature redundancy, and selected the optimal feature group as the input of SVM. The second one was a well-known CNN-based method (ResNet [30]) where we directly used the obtained MIP images to train a ResNet model. The third one was a pure Transformer-based method (MViTv2 [31]) whose input was also consistent with MPViT. For fair comparison, all four methods applied the same preprocessing steps and data augmentation.

To better evaluate the performance for collateral scoring, we used five-fold cross-validation for all four methods. In each cross-validation experiment, all 154 patients were randomly divided into five folds, and then we used four folds for training and parameter tuning, and used the remaining one fold for testing the trained model. After repeating the above operation five times, we obtained the collateral scores of all 154 patients.

### 2.4. Statistical Analysis

For evaluating the performance of collateral scoring, the agreement between automated collateral scores and expert-reading collateral scores was analyzed by intraclass correlation coefficients (ICC), as well as Kappa and the Pearson correlation coefficient. To further assess the clinical significance of the model, accuracy, sensitivity, area under the receiver operating characteristic curve (AUC) and specificity were used to evaluate the binary-classification performance. We calculated the 95% confidence interval (CI) for each evaluation metric. In the poor vs. non-poor classification, we considered patients with poor collateral and non-poor (good or intermediate) collateral as positive and negative samples, respectively. In the good vs. non-good classification, we considered patients with good collateral and non-good (poor or intermediate) collateral as positive and negative samples, respectively. In addition, subgroup analyses were performed to find the relationship between the collateral scoring performance and some clinical factors including gender, age, Alberta Stroke Program Early CT score (ASPECTS), National Institute of Health Stroke scale (NIHSS) and time from onset to CT imaging.

Accuracy, precision, sensitivity, AUC, specificity and kappa were calculated using SciKit learn toolkit. Pearson coefficient was calculated using the Scipy toolkit, and ICC was calculated using the Pingouin toolkit. The Z-test based on Fisher Z-transformation was used to test whether there were significant differences between the ICC, Kappa and Pearson values of two methods. The N-1 Chi-squared test was used to test whether there were significant differences between the accuracy, sensitivity and specificity of two methods. Statistics tests were performed with software (MedCalc, version 20.0.3, MedCalc Software, Mariakerke, Belgium). A *p*-value smaller than 0.05 was considered to represent a significant difference.

## 3. Results

### 3.1. Study Participants

Table 1 lists the patient characteristics of the 154 patients involved in this study. The median age was 72 years (interquartile range [IQR], 64–80 years). Out of the 154 patients, 91 patients were male. Median onset-to-CT time was 135 min (IQR, 90–240 min). Median follow-up infarct volume was 4.6 mL (IQR, 0.6–26.0 mL). Median baseline NIHSS, modified Rankin scale (mRS) and ASPECTS were 11.5 (IQR, 4–21), 0 (IQR, 0–1) and 10 (IQR, 9–10), respectively. Median 90 day mRS was 2 (IQR, 1–4).

### 3.2. Results for Collateral Scoring

#### 3.2.1. Three-Point Collateral Score Classification

**Figure 4:**
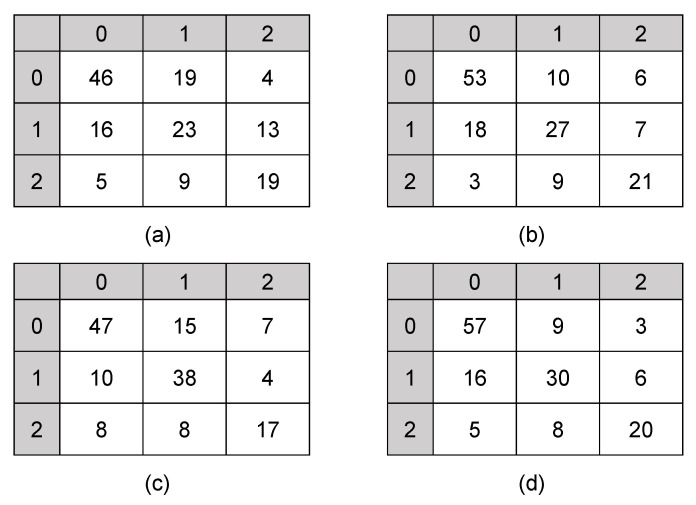
Confusion matrix between automated scores versus consensus scores. (**a**) SVM; (**b**) ResNet; (**c**) MViTv2; (**d**) MPViT.

To show the superiority of MPViT (a hybrid CNN and Transformer network), we compared it with three different automated collateral scoring methods: SVM, ResNet and MViTv2. Table 2 details the comparisons of the four automated scoring methods. Figure 4 shows the confusion matrix between the collateral scores obtained by the four methods and the expert consensus scores.

MPViT achieved an ICC of 0.767 (95% CI: 0.68–0.83), a Kappa of 0.6184 (95% CI: 0.4954–0.7414) and a Pearson of 0.6621 (95% CI: 0.5112–0.7068), which were all better than those of the three other types of methods: SVM, ResNet and MViTv2. From Figure 4, we found that MPViT achieved a good tradeoff for the three classes of collateral scores. These results indicate that MPViT had better agreement between the scores obtained by automated methods and the expert scores than traditional machine learning methods, pure convolutional models and pure Transformer models. The reason might be that MPViT combines the advantages of both CNN and Transformer to capture local and global representations for collateral scoring.

#### 3.2.2. Results for Poor vs. Non-Poor and Good vs. Non-Good Classification

In addition to three-point score classification, a dichotomized collateral score might be more useful in decision making.

Table 3 and Table 4 show the performance of the four compared methods for poor vs. non-poor and good vs. non-good classification. For poor vs. non-poor classification, MPViT achieved an accuracy of 85.71% (95% CI: 80.18–91.24%), an AUC of 0.766 (95% CI: 0.691–0.83) and a specificity of 92.56% (95% CI: 86.3–96.5%), outperforming the other three methods regarding most metrics. For good vs. non-good classification, MPViT achieved an accuracy of 78.57% (95% CI: 72.09–85.05%), an AUC of 0.79 (95% CI: 0.717–0.851) and a specificity of 82.60% (95% CI: 71.6–90.7%), which is higher than those of the other three methods.

#### 3.2.3. Subgroup Analysis Based on Patient Characteristics

In this section, we further explore whether there were clinical factors that were related to the performance of collateral scoring via subgroup analysis (Table 5).

We first used gender and age to stratify all 154 patients into subgroups: male patients and female patients, and patients ≥ 70 years old and patients < 70 years old. The results in Table 5 sho that MPViT achieved an ICC of 0.8716 (95% CI: 0.7878–0.9223), a Kappa of 0.7718 (95% CI: 0.6191–0.9245) and a Pearson of 0.7728 (95% CI: 0.6494–0.8552) on female patients, which were significantly better than the performance on male patients (ICC: 0.6549 [95% CI: 0.4771–0.7723], *p* = 0.0009; Kappa: 0.475 [95% CI: 0.2973–0.6527], *p* = 0.0024; Pearson: 0.489 [95% CI: 0.3045–0.6160], *p* = 0.0033), which indicated that the female patients’ scoring results had better reliability. The performance of MPViT on patients ≥ 70 years old was non-significantly better than the performance on patients < 70 years old (P values of all metrics >0.05), which shows that the older patients were scored better.

Second, we also stratified patients by clinical scores including baseline ASPECTS (≥7 vs. <7) and NIHSS (≥9 vs. <9) for subgroup analysis according to [32,33]. For the ASPECTS subgroups, the automated scores had non-significantly better agreement with the expert manual scores on patients with ASPECTS ≥ 7. Studies have shown that patients with ASPECTS scores greater than 7 could benefit significantly from mechanical thrombectomy [32]; thus, MPViT has potential to select suitable AIS patients for mechanical thrombectomy. For the NIHSS subgroups, ICC on patients with NIHSS < 9 (0.7308 [95% CI: 0.4165–0.7009]) was significantly better than ICC (0.526 [95% CI: 0.2289–0.7087]) on patients with NIHSS ≥ 9 (*p* = 0.0371) and other metrics on patients with NIHSS < 9 were better but not significantly so (all *p* > 0.05).

According to the onset-to-CT time, we divided all patients into two subgroups of less than 180 min and ≥ 180 min for subgroup analysis. The ICC, Kappa and Pearson on patients with onset-to-CT time < 180 min were all non-significantly higher than those on the other subgroup. This implies that the earlier the CT scans were scanned, the better the collateral scoring performance was.

## 4. Discussion

In this study, we explored several automated collateral scoring methods without vessel segmentation on CTA images that combined the advantages of CNNs and Transformers. Our results show the superiority of hybrid CNN and Transformer models for both three-point collateral scoring and dichotomized scoring, i.e., poor vs. non-poor and good vs. non-good collateral scores.

Comparisons showed that the hybrid CNN and Transformer model—the MPViT based vessel-segmentation free method—achieved better performance than radiomics and traditional machine learning-based, pure CNN-based and pure Transformer-based models for three-point collateral scoring. MPViT also achieved better performance for poor vs. non-poor and good vs. non-good classification. These results also confirm our previous hypothesis that the hybrid CNN and Transformer model could achieve better collateral scoring, showing that combining CNN and Transformer is promising, and showing that the designed MPViT could provide support information for clinical decision-making. Additionally, these results also validate that vessel-segmentation free methods for collateral scoring are feasible. This study could provide future directions for designing good collateral scoring methods.

The average time including preprocessing and classification using the trained model for collateral scoring of a patient was 8.26 s per patient, which is obviously less than the time (more than 5 min) needed for radiologist to score the collateral of a patient. Therefore, the proposed method could reduce the time of collateral scoring and decrease the burden of doctors for diagnosis and prognosis of AIS, and then has potential to reduce the economic cost of AIS patients. Additionally, it can be seen from the results that the proposed automated collateral scoring model had a high enough consistency with total scores read by experts and especially achieved better performance for the binary classification tasks such as good and non-good classification, which makes the proposed method helpful for selecting patients who could achieve good prognosis. In summary, due to the efficiency and the effectiveness of the proposed method for collateral scoring, it is feasible to be applied in the AIS clinical context to diagnose AIS and make treatment decisions.

When stratifying the patients based on clinical characteristics, we found that the clinical factors of gender, age, baseline ASPECTS, baseline NIHSS and onset-to-CT time were all related to collateral scoring, and gender was the most significant related factor to collateral scoring. These findings imply that incorporating these clinical factors into the automated collateral scoring models might potentially further improve the performance.

This study has several limitations. The sample size in this paper was limited, and there was no external validation cohort. More patients will be collected to obtain more effective models and test the generalizability of the designed models. Second, the timing of CTA acquisition is not accounted for in our model. However, around 10–20% of CTA scans were not acquired at the peak artery phase, which might bias the evaluation results. Multi-phase CTA scans can provide temporal information, and have potential to achieve more reliable collateral scoring across different observers. Extending our current models on multi-phase CTA scans should be explored in the future.

## 5. Conclusions

This study explored a vessel segmentation free collateral scoring method, i.e., hybrid CNN and Transformer model—MPViT—on single-phase CTA scans of AIS patients. The results show that the hybrid CNN and Transformer model, MPViT, could achieve accurately automated three-point collateral scoring and dichotomized collateral scoring. Subgroup analysis revealed that clinical factors, such as gender, age, baseline ASPECTS, baseline NIHSS and onset-to-CT time, were associated with collateral scoring. This study provides evidence that artificial intelligence is helpful to objectively assess collateral status based on imaging, therefore assisting decision making.

## Figures and Tables

**Table 1:** Patient Characteristics for all 154 AIS patients collected.

Characteristics	All 154 Patients
Median age, years (IQR)	72 (64–80)
Gender, male, No. (%)	91 (59)
Median onset-to-CT time (IQR), min	135 (90–240)
Median follow-up infarct volume (IQR), mL	4.6 (0.6–26.0)
Median baseline NIHSS (IQR)	11.5 (4–21)
Median baseline mRS (IQR)	0 (0–1)
Median baseline ASPECTS(IQR)	10 (9–10)
Median 90 days mRS (IQR)	2 (1–4)

**Table 2:** Performance for three-point collateral score classification.

Method	ICC	Kappa	Pearson
SVM	0.6774 [0.56, 0.77]	0.5118 [0.3842, 0.6393]	0.5123 [0.3854, 0.6194]
ResNet	0.7358 [0.64, 0.81]	0.5818 [0.4555, 0.7080]	0.5822 [0.4671, 0.6773]
MViTv2	0.6246 [0.48, 0.73]	0.4541 [0.2985, 0.6097]	0.4548 [0.3196, 0.5707]
MPViT	0.767 [0.68, 0.83]	0.6184 [0.4954, 0.7414]	0.6621 [0.5112, 0.7068]

**Table 3:** Performance for poor vs. non-poor classification.

Method	Accuracy (%)	Sensitivity (%)	AUC	Specificity (%)
SVM	79.87 [73.53, 86.2]	57.57 [39.2, 74.5]	0.718 [0.64, 0.787]	85.95 [78.5, 91.6]
ResNet	83.76 [77.94, 89.59]	63.63 [45.1, 79.6]	0.764 [0.689, 0.829]	89.25 [82.3, 94.2]
MViTv2	82.46 [76.46, 88.47]	51.51 [33.5, 69.2]	0.712 [0.634, 0.782]	90.9 [84.3, 95.4]
MPViT	85.71 [80.18, 91.24]	60.6 [42.1, 77.1]	0.766 [0.691, 0.83]	92.56 [86.3, 96.5]

**Table 4:** Performance for good vs. non-good classification.

Method	Accuracy (%)	Specificity (%)	AUC	Sensitivity (%)
SVM	71.42 [64.29, 78.56]	75.29 [64.7, 84.0]	0.71 [0.631, 0.78]	66.66 [54.3, 77.6]
ResNet	75.97 [69.22, 82.72]	75.29 [64.7, 84.0]	0.761 [0.685, 0.826]	76.81 [65.1, 86.1]
MViTv2	74.25 [67.1, 80.95]	78.82 [68.6, 86.9]	0.735 [0.658, 0.803]	68.11 [55.8, 78.8]
MPViT	78.57 [72.09, 85.05]	75.29 [64.7, 84.0]	0.79 [0.717, 0.851]	82.6 [71.6, 90.7]

**Table 5:** Three-point collateral scoring performance of MPViT of subgroups stratified by gender, age, ASPECTS, NIHSS and onset-to-CT time. * Denotes significant difference (*p* < 0.05).

Variable	Subgroup	ICC	Kappa	Pearson
Gender	male	0.6549 [0.4771, 0.7723]	0.475 [0.2973, 0.6527]	0.489 [0.3045, 0.6160]
	female	0.8716 * [0.7878, 0.9223]	0.7718 * [0.6191, 0.9245]	0.7728 * [0.6494, 0.8552]
Age (years)	≥70	0.7729 [0.6542, 0.8509]	0.6294 [0.4714, 0.7873]	0.6303 [0.4859, 0.7399]
	<70	0.6732 [0.4643, 0.8007]	0.4921 [0.2648, 0.7195]	0.519 [0.2966, 0.6482]
Baseline ASPECTS	≥7	0.7497 [0.6493, 0.8215]	0.5986 [0.4624, 0.7347]	0.5997 [0.4798, 0.6960]
	<7	0.7636 [0.3473, 0.9144]	0.5233 [0.2264, 0.8202]	0.7 [0.2129, 0.7379]
Baseline NIHSS	≥9	0.7308 * [0.4165, 0.7009]	0.5392 [0.3792, 0.6993]	0.5801 [0.3840, 0.6649]
	<9	0.526 [0.2289, 0.7087]	0.3383 [0.0933, 0.5833]	0.3897 [0.1420, 0.5091]
Onset-to-CT time (min)	≥180	0.7245 [0.5408, 0.8347]	0.5592 [0.3349, 0.7835]	0.5699 [0.3638, 0.7075]
	<180	0.7888 [0.6807, 0.8604]	0.6498 [0.5146, 0.7535]	0.6515 [0.5146, 0.7535]

## Data Availability

The data presented in this study are available upon request from the corresponding author.

## Abbreviations

The following abbreviations are used in this manuscript:

AIS	Acute ischemic stroke
ASPECTS	Alberta Stroke Program Early CT score
CTA	Computed tomography angiography
CNN	Convolutional neural network
ICC	Intraclass correlation coefficient
MIP	Maximum intensity projection
AUC	Area under the receiver operating characteristic curve
NIHSS	National Institute of Health Stroke scale
IQR	interquartile range
mRS	modified Rankin scale
